# A Coordinated Adhesion-Molecule Activation Profile in Pediatric Sepsis: A Prospective Cohort Study from Vietnam

**DOI:** 10.3390/pediatric18030078

**Published:** 2026-06-09

**Authors:** Bui Thanh Liem, Chu Van Thien, Nguyen Trong Nghia, Le Anh Phong, Ngo Nhu Dinh, Nguyen Huy Luan, Phung Nguyen The Nguyen

**Affiliations:** 1 Department of Pediatrics, School of Medicine, University of Medicine and Pharmacy at Ho Chi Minh City, Ho Chi Minh City 700000, Vietnam; 2 Department of Pediatric Intensive Care, Children’s Hospital 2, Ho Chi Minh City 700000, Vietnam; 3 Dong Nai Children’s Hospital, Dong Nai City 760000, Vietnam

**Keywords:** pediatric sepsis, soluble adhesion molecules, endothelial activation, leukocyte adhesion, soluble vascular cell adhesion molecule-1, L-selectin, C-reactive protein, immune–vascular dysregulation, Vietnam

## Abstract

**Background/Objectives:** Pediatric sepsis is increasingly recognized as a syndrome involving immune–vascular dysregulation. However, most pediatric biomarker studies focus on individual molecules rather than coordinated patterns of leukocyte–endothelial activation. This study aimed to evaluate whether children diagnosed with sepsis within 48 h of admission showed a coordinated soluble adhesion-molecule activation profile measured at enrollment. **Methods:** This prospective cohort study included 144 children aged 1–60 months with suspected infection enrolled at Dong Nai Children’s Hospital, Vietnam, from May 2021 to October 2022. Blood samples were collected at enrollment. Sepsis was classified according to the 2005 International Pediatric Sepsis Consensus Conference (IPSCC) criteria within 48 h of admission. Twelve soluble adhesion molecules were measured using a multiplex immunoassay. A composite adhesion activation score was derived by log2 transformation, z-score standardization, and averaging across the 12 markers. Principal component analysis (PCA) was used as an exploratory method to summarize the shared variation across the adhesion-molecule panel. C-reactive protein (CRP) was included as a routinely available inflammatory comparator. **Results:** Among 144 children, 32 (22.2%) were diagnosed with sepsis within 48 h of admission. Individual marker discrimination was strongest for L-selectin (area under the receiver operating characteristic curve [AUC] 0.883), followed by soluble vascular cell adhesion molecule-1 (sVCAM-1; AUC 0.855), intercellular adhesion molecule-3 (ICAM-3; AUC 0.838), P-selectin glycoprotein ligand-1 (PSGL-1; AUC 0.836), E-selectin (AUC 0.819), and intercellular adhesion molecule-2 (ICAM-2; AUC 0.819). CRP also differed between children with and without sepsis but had a lower AUC than the leading adhesion molecules in descriptive ROC analyses. The composite adhesion activation score was strongly associated with sepsis (odds ratio 7.95 per 1-standard deviation increase; 95% confidence interval 3.44–18.40; *p* < 0.001) and showed good discrimination (AUC 0.855; 95% confidence interval 0.776–0.931). The first principal component explained 70.0% of biomarker variance, consistent with coordinated elevation of correlated adhesion molecules. **Conclusions:** In this prospective Vietnamese pediatric cohort, children diagnosed with sepsis within 48 h of admission showed coordinated elevation of soluble adhesion molecules measured at enrollment. These findings support the biological relevance of leukocyte–endothelial activation in pediatric sepsis. However, the adhesion-molecule activation profile should be considered exploratory and hypothesis-generating, requiring external validation and further evaluation against simplified, clinically feasible biomarker approaches.

## 1. Introduction

Sepsis in children remains a major cause of preventable morbidity and mortality worldwide, particularly in low- and middle-income countries where delayed recognition, limited laboratory access, and constrained critical care resources can worsen outcomes [1,2,3]. Early identification is challenging because fever, tachycardia, tachypnea, poor feeding, and altered activity overlap with uncomplicated infections. Routinely available inflammatory markers, such as C-reactive protein (CRP), may support clinical assessment but do not directly capture the immune–vascular processes that link infection to microvascular dysfunction and organ injury.

The vascular endothelium is a central effector in sepsis pathobiology [4,5]. During infection, inflammatory cytokines, pathogen-associated molecular patterns, complement activation, and coagulation perturbation drive endothelial activation, barrier dysfunction, leukocyte trafficking, platelet–endothelial interaction, and microvascular injury. Soluble adhesion molecules are measurable circulating correlates of these processes and may reflect endothelial activation, leukocyte–endothelial interaction, platelet activation, and intercellular adhesion [5,6,7,8].

Most pediatric sepsis biomarker studies evaluate individual analytes and their diagnostic performance. However, sepsis is unlikely to be represented by a single molecule. A coordinated soluble adhesion-molecule activation profile may provide additional biological insight into immune–vascular activation during pediatric infection. Beyond classic endothelial markers such as soluble vascular cell adhesion molecule-1 (sVCAM-1), soluble intercellular adhesion molecule-1 (sICAM-1), E-selectin, and P-selectin, broader adhesion pathways involving L-selectin, P-selectin glycoprotein ligand-1 (PSGL-1), intercellular adhesion molecule-2 (ICAM-2), intercellular adhesion molecule-3 (ICAM-3), platelet/endothelial cell adhesion molecule-1 (PECAM-1), cluster of differentiation 44 (CD44), epithelial cell adhesion molecule (EpCAM), and neural cell adhesion molecule (NCAM) may provide insight into leukocyte trafficking and multicellular immune–vascular activation [9,10,11].

Data on soluble adhesion-molecule profiles in pediatric sepsis from Southeast Asia remain limited. We therefore analyzed a prospective Vietnamese cohort of children with suspected acute infection requiring inpatient management using a 12-marker soluble adhesion-molecule panel measured at enrollment. We aimed to evaluate whether children diagnosed with sepsis within 48 h of admission showed coordinated elevation of soluble adhesion molecules, to derive an exploratory composite adhesion activation score, and to summarize shared biomarker variation using principal component analysis (PCA). CRP was included as a routinely available inflammatory comparator because of its practical relevance in low-resource clinical settings.

## 2. Materials and Methods

### 2.1. Study Design and Setting

This was a prospective observational cohort study conducted at Dong Nai Children’s Hospital, Dong Nai Province, Vietnam. Young children aged 1–60 months were enrolled from May 2021 to October 2022. The study was approved by Biomedical Research Ethics Committee, University of Medicine and Pharmacy at Ho Chi Minh City (IRB-VN01002/IRB00010293/FWA00023448). Written informed consent was obtained from parents or legal guardians before enrollment.

### 2.2. Participants, Suspected Infection, and Sepsis Classification

Children presenting to the outpatient clinic or emergency department with suspected acute infection and subsequently requiring inpatient management were eligible. Children were excluded if trauma or injury was the primary reason for presentation; if they presented within 72 h after vaccination; if they had known chronic conditions expected to substantially affect the inflammatory response or clinical course, including immunodeficiency, malignancy, HIV infection, thalassemia, prolonged corticosteroid therapy, active chronic infections such as tuberculosis or hepatitis B, or active chronic cardiopulmonary disease; if they had been hospitalized overnight at any healthcare facility during the current illness before presentation to the study site; if they had previously been enrolled in the study during an earlier illness episode; or if they had received more than 15 min of inpatient treatment before completion of the study enrollment procedures. Suspected infection was defined clinically by the treating physician in children presenting with acute fever or hypothermia and symptoms or signs suggestive of an infectious illness. Microbiological confirmation was not required for inclusion, reflecting the early clinical assessment setting in which treatment and hospitalization decisions were made.

Patients were included in the present analysis if clinical data and an enrollment blood sample were available. Blood samples for biomarker measurement were collected at enrollment, before biomarker results were available to clinicians. Sepsis was classified according to the 2005 International Pediatric Sepsis Consensus Conference (IPSCC) criteria, which define pediatric sepsis as systemic inflammatory response syndrome in the presence of suspected or proven infection [1]. For this analysis, sepsis status was determined within 48 h of admission. Biomarker results were not used to define sepsis. Children who had suspected acute infection requiring inpatient management but did not meet IPSCC sepsis criteria within 48 h were included as the non-sepsis comparator group. This comparator group therefore represented clinically ill children with suspected infection rather than healthy controls.

Blood cultures and organism-level microbiological testing were not systematically obtained as part of the study protocol. In routine care, blood cultures were performed only when clinically indicated by the treating physicians. Because microbiological testing was not standardized or recorded as a study variable, isolated organisms and culture-confirmed infection status were not available for reliable analysis.

### 2.3. Biomarker Measurement

Blood samples were collected in K3-EDTA tubes at enrollment. EDTA plasma was separated, aliquoted, and stored frozen until biomarker analysis. Soluble adhesion molecules were quantified using the LEGENDplex™ Human Adhesion Molecule Panel (13-plex) with V-bottom Plate, Catalog No. 740946, Lot No. B472821, BioLegend, Inc. (a Revvity Company), San Diego, CA, USA, a bead-based multiplex sandwich immunoassay read by flow cytometry. The panel included sICAM-1, ICAM-2, ICAM-3, sVCAM-1, PECAM-1, ALCAM, EpCAM, NCAM, E-selectin, P-selectin, L-selectin, PSGL-1, and CD44; 12 markers were included in the present analysis. Plasma samples were diluted 1:2 using Assay Buffer and assayed according to the manufacturer’s instructions. Data were acquired on a NovoCyte D2060 flow cytometer (Agilent Technologies, Santa Clara, CA, USA) and analyzed using LEGENDplex™ Data Analysis Software version 8.0 (BioLegend, Inc., San Diego, CA, USA) with 5-parameter logistic curve fitting. Final concentrations were adjusted for the dilution factor and converted to ng/mL for reporting. All 144 samples were analyzed using the same kit lot to minimize inter-lot variability.

CRP was included as a routinely available inflammatory marker for comparison with soluble adhesion molecules. CRP was measured from blood samples collected at enrollment, at the same time as the soluble adhesion-molecule biomarkers, according to routine hospital laboratory procedures.

### 2.4. Data Preprocessing, Composite Adhesion Activation Score, and PCA

Biomarker distributions were right-skewed and were summarized as medians with interquartile ranges. For log-transformed analyses, each biomarker was transformed using log2 concentration. Values equal to zero were considered below the assay reporting threshold and were replaced by one-half of the minimum positive value for that biomarker before log2 transformation. Log2-transformed markers were then standardized to z-scores.

A composite adhesion activation score was calculated as the mean of the 12 standardized log2-transformed biomarker values, with higher scores indicating greater overall soluble adhesion-molecule activation. Equal weighting was used to avoid data-driven weighting in this modest-sized cohort and to provide a transparent summary measure of overall adhesion-molecule activation. The performance of individual markers was therefore reported separately to avoid implying that all markers contributed equally to discrimination. This score was used as an exploratory summary measure of coordinated adhesion-molecule elevation across the panel.

PCA was performed on the matrix of standardized log2-transformed biomarker concentrations to summarize shared variation across the soluble adhesion-molecule panel. The first principal component (PC1) was oriented such that higher values represented higher adhesion-molecule activation. PC1 was interpreted as an exploratory adhesion-molecule activation axis rather than a validated biological subtype. For descriptive analyses, patients were categorized into low, intermediate, and high activation groups according to tertiles of the composite adhesion activation score. Because the analytic cohort included 144 children, each tertile contained 48 patients.

### 2.5. Statistical Analysis

Continuous variables were presented as median (interquartile range) and compared using the Mann–Whitney U test or Kruskal–Wallis test as appropriate. Categorical variables were presented as number (percentage) and compared using Fisher’s exact test or chi-square test. Receiver operating characteristic (ROC) curves and the area under the ROC curve (AUC) were used for discrimination analyses; bootstrap resampling was used to estimate 95% confidence intervals. Logistic regression was used to estimate odds ratios for sepsis per 1-standard-deviation increase in the composite adhesion activation score and PC1. Two-sided *p* values less than 0.05 were considered statistically significant. Analyses were performed using Python version 3.11.9 (Python Software Foundation, Wilmington, DE, USA) and R version 4.3.2 (R Foundation for Statistical Computing, Vienna, Austria).

The primary analysis evaluated the association between the composite 12-marker adhesion activation score and sepsis diagnosed within 48 h of admission. Secondary analyses included individual marker discrimination, PCA-derived PC1 analysis, comparison with CRP as a routinely available inflammatory marker, and comparison of clinical characteristics across tertile-defined adhesion activation groups. All composite score and PCA analyses were considered exploratory. No formal a priori sample size calculation was performed for the present exploratory biomarker analysis. The analytic sample size was determined by the number of enrolled children with available clinical data and sufficient stored plasma for multiplex adhesion-molecule measurement. Therefore, all composite score and PCA analyses should be interpreted as exploratory.

### 2.6. Reporting Guidelines

This manuscript was prepared in accordance with relevant STROBE recommendations for observational cohort studies [12]. Diagnostic discrimination analyses were reported with reference to STARD principles where applicable [13].

## 3. Results

### 3.1. Study Population

The final analytic cohort included 144 children aged 1–60 months with suspected acute infection requiring inpatient management. Blood samples for biomarker measurement were collected at enrollment. Sepsis was classified within 48 h of admission according to the 2005 International Pediatric Sepsis Consensus Conference (IPSCC) criteria. Overall, 32 children (22.2%) were diagnosed with sepsis within 48 h, whereas 112 children (77.8%) were included in the non-sepsis comparator group. The non-sepsis group consisted of clinically ill children with suspected acute infection who required inpatient management but did not meet IPSCC sepsis criteria within 48 h.

Baseline clinical and laboratory characteristics are summarized in Table 1. Children with sepsis had higher lactate concentrations and were more frequently admitted to the intensive care unit (ICU). C-reactive protein (CRP), included as a routinely available inflammatory marker, was also higher in children with sepsis than in those without sepsis. No deaths occurred in the analytic cohort. Blood cultures were not systematically obtained as part of the study protocol, and organism-level microbiological data were therefore not available for reliable analysis.

### 3.2. Adhesion Molecule Concentrations by Sepsis Status

Children diagnosed with sepsis within 48 h had higher circulating concentrations of multiple soluble adhesion molecules measured at enrollment (Table 2). This pattern was consistent with coordinated elevation of markers related to leukocyte–endothelial interaction, endothelial activation, and intercellular adhesion.

### 3.3. Individual Marker Discrimination and Comparison with CRP

In receiver operating characteristic analyses, the leading individual adhesion markers were L-selectin, sVCAM-1, ICAM-3, PSGL-1, E-selectin, and ICAM-2 (Table 3). L-selectin showed the highest individual discriminatory performance for sepsis within 48 h. The composite adhesion activation score and the PCA-derived first principal component (PC1) also showed good discrimination, but they did not clearly outperform the best individual marker. CRP had a lower AUC than the leading adhesion molecules in descriptive ROC analyses, although it was significantly higher in children with sepsis; no formal statistical comparison between ROC curves was performed.

### 3.4. Composite Adhesion Activation Score and PCA-Derived Activation Axis

**Figure 1:**
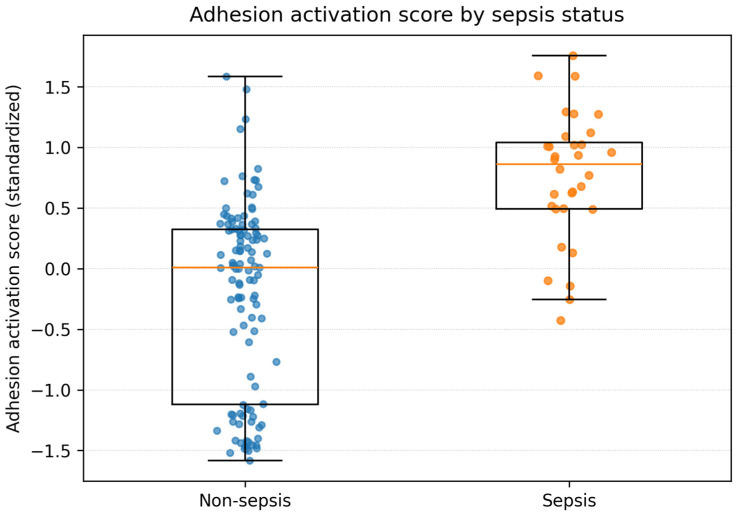
Composite 12-marker adhesion activation score by sepsis status. The box represents the interquartile range, the horizontal line indicates the median, whiskers indicate the range within 1.5 times the interquartile range, and individual points represent participants.

The composite 12-marker adhesion activation score was higher in children diagnosed with sepsis within 48 h than in those without sepsis (Figure 1) and was strongly associated with sepsis (Table 4). Each 1-standard deviation increase in the composite score was associated with an odds ratio of 7.95 for sepsis (95% confidence interval, 3.44–18.40; *p* < 0.001). The score showed good discrimination, with an AUC of 0.855.

PCA was performed using the standardized log2-transformed concentrations of the 12 soluble adhesion molecules. PC1 explained 70.0% of the total biomarker variance. The directionally consistent loadings across markers supported the presence of shared variation across the adhesion-molecule panel, consistent with coordinated elevation of correlated adhesion molecules. PC1 was also strongly associated with sepsis and showed similar discrimination to the composite score. These PCA findings were interpreted as exploratory and were not considered to define a validated biological subtype.

Detailed principal component loadings, supporting correlation analyses, and sensitivity analyses excluding EpCAM and PSGL-1 are provided in the Supplementary Materials.

### 3.5. Clinical Features Across Tertile-Defined Adhesion Activation Groups

**Figure 2:**
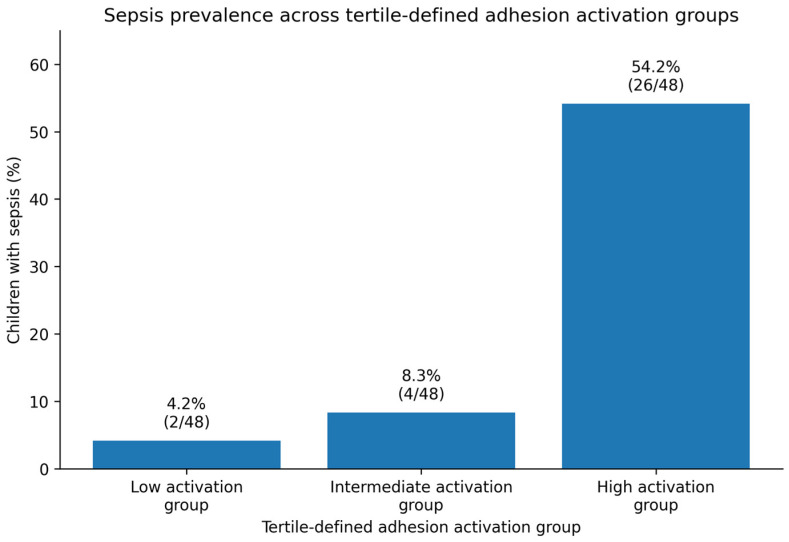
Proportion of children with sepsis across tertile-defined low, intermediate, and high adhesion activation groups. Each activation group contained 48 children.

For descriptive analyses, the cohort was divided into low, intermediate, and high adhesion activation groups according to tertiles of the composite adhesion activation score. Each group contained 48 children. The prevalence of sepsis increased across tertiles, from 2 of 48 children (4.2%) in the low activation group to 4 of 48 children (8.3%) in the intermediate activation group and 26 of 48 children (54.2%) in the high activation group (*p* < 0.001, Figure 2). These groups should be interpreted as exploratory activation strata derived within this cohort, rather than externally validated biological phenotypes.

Clinical and laboratory characteristics across activation groups are shown in Table 5. Most baseline vital signs and demographic variables were broadly similar across groups. White blood cell count differed across activation groups, with higher values in the intermediate and high activation groups than in the low activation group (*p* = 0.031). C-reactive protein (CRP) was numerically higher in the high activation group than in the low and intermediate groups, although the overall difference across tertiles did not reach statistical significance (*p* = 0.066). Lactate concentrations and intensive care unit admission did not differ significantly across activation groups.

Overall, these findings suggest that higher composite adhesion activation was associated with a greater proportion of children classified as having sepsis within 48 h of admission. However, the tertile-defined activation groups were not consistently associated with all available clinical severity indicators. Therefore, the activation group analysis should be considered exploratory and primarily descriptive.

## 4. Discussion

In this prospective Vietnamese pediatric cohort, children diagnosed with sepsis within 48 h of admission showed coordinated elevation of multiple soluble adhesion molecules measured at enrollment. This finding supports the biological relevance of leukocyte–endothelial and immune–vascular activation in pediatric sepsis. The composite 12-marker adhesion activation score and the principal component analysis (PCA)-derived activation axis were both strongly associated with sepsis and showed good discriminatory performance. However, these measures should be interpreted as exploratory summaries of correlated adhesion-molecule elevation within this cohort, rather than as validated biological phenotypes or clinical endotypes.

The biological interpretation of these findings is consistent with contemporary understanding of sepsis as a syndrome involving immune–vascular dysregulation [4,14,15,16]. The vascular endothelium is not merely a passive barrier but an active regulator of inflammation, vascular permeability, coagulation, leukocyte trafficking, and microvascular homeostasis [4,5]. During sepsis, pathogen-associated molecular patterns, inflammatory cytokines, complement activation, coagulation perturbation, and tissue injury signals may promote endothelial activation and dysfunction. These processes contribute to leukocyte recruitment, capillary leak, platelet–endothelial interaction, microvascular injury, and ultimately organ dysfunction [4,14,15,16].

Soluble adhesion molecules provide measurable circulating correlates of these immune–vascular processes [5,6,7,8]. Soluble vascular cell adhesion molecule-1 (sVCAM-1), soluble intercellular adhesion molecule-1 (sICAM-1), selectins, P-selectin glycoprotein ligand-1 (PSGL-1), platelet/endothelial cell adhesion molecule-1 (PECAM-1), cluster of differentiation 44 (CD44), epithelial cell adhesion molecule (EpCAM), and neural cell adhesion molecule (NCAM) represent overlapping but non-identical aspects of leukocyte trafficking, endothelial adhesion, platelet–leukocyte interaction, and intercellular communication [5,6,7,8,9,10,11]. The high inter-marker correlations and the finding that the first principal component explained approximately 70% of biomarker variance suggest shared variation across the adhesion-molecule panel, consistent with coordinated elevation of correlated soluble adhesion molecules in children with sepsis.

A notable finding was that L-selectin showed the strongest individual discrimination, followed by sVCAM-1, intercellular adhesion molecule-3 (ICAM-3), PSGL-1, E-selectin, and intercellular adhesion molecule-2 (ICAM-2). This pattern broadens the interpretation beyond endothelial activation alone and suggests involvement of both endothelial and leukocyte-associated adhesion pathways. L-selectin and PSGL-1 are central to leukocyte rolling and recruitment, whereas ICAM family members and VCAM-1 contribute to firm adhesion and leukocyte–endothelial interaction [9,10,11]. These findings are therefore best interpreted as evidence of coordinated adhesion-molecule activation in children with sepsis, rather than isolated elevation of a single endothelial marker.

Importantly, the composite adhesion activation score and the PCA-derived activation axis did not clearly outperform the strongest individual marker. Their value in the present study is therefore primarily biological and descriptive: they summarize coordinated variation across the adhesion-molecule panel and support the concept that multiple leukocyte–endothelial pathways are activated together in pediatric sepsis. These findings should not be interpreted as establishing a clinically validated phenotype, biological endotype, or deployable diagnostic test. Although sepsis phenotyping is an important and evolving research direction [17], the present study was not designed to define or validate sepsis endotypes. External validation is required before these exploratory biomarker profiles can be used for risk stratification or clinical decision-making.

C-reactive protein (CRP) was included as a routinely available inflammatory comparator because it is inexpensive, widely used, and more feasible than multiplex biomarker panels in low- and middle-income settings [2]. In this cohort, CRP was higher in children with sepsis and showed discriminatory ability. Its AUC was descriptively lower than that of the leading adhesion molecules, but this comparison should be interpreted cautiously because formal statistical comparisons between ROC curves were not performed. This finding suggests that soluble adhesion molecules may capture immune–vascular information not directly reflected by CRP. Nevertheless, CRP remains more clinically accessible, and the practical value of adhesion markers will depend on whether simplified biomarker approaches can provide incremental information beyond routinely available clinical and laboratory assessment.

The clinical implementation of a 12-marker soluble adhesion-molecule panel remains uncertain, particularly in low- and middle-income settings. Multiplex testing requires specialized laboratory infrastructure, standardized sample handling, quality control, and acceptable turnaround time. Therefore, the present findings should be considered hypothesis-generating rather than practice-changing. A more feasible translational pathway may be to identify a simplified marker strategy, potentially based on the strongest-performing individual markers such as L-selectin and sVCAM-1, and to evaluate whether such a simplified approach improves risk assessment beyond CRP and clinical evaluation.

This study has several strengths. It was prospective, enrolled young children with suspected acute infection in a real-world Vietnamese pediatric setting, and measured a broad soluble adhesion-molecule panel from enrollment samples. The cohort also used clinically ill children with suspected infection as the comparator group rather than healthy controls. This design makes the biomarker comparisons more clinically relevant to early hospital assessment, because the non-sepsis group represented children with suspected acute infection requiring inpatient management who did not meet sepsis criteria within 48 h.

Several limitations should be acknowledged. First, this was a single-center study with a modest number of sepsis cases, so external validation is required. Because this cohort included children aged 1–60 months from a single Vietnamese pediatric hospital, the findings may not be generalizable to neonates, older children, or populations with different infectious etiologies and case-mix. Second, sepsis classification used the 2005 International Pediatric Sepsis Consensus Conference criteria [1]. These criteria remain historically important and were appropriate for the study period, but they are based on systemic inflammatory response in the presence of suspected or proven infection [1]. Contemporary definitions, including the 2024 Phoenix criteria, emphasize organ dysfunction more directly [3]. Because full organ dysfunction variables required for Phoenix classification were not systematically available, retrospective reclassification according to contemporary organ dysfunction-based criteria was not feasible. Accordingly, the observed adhesion-molecule activation profile may partly reflect systemic inflammatory activation captured by IPSCC/SIRS-based classification, rather than pediatric sepsis as defined by contemporary organ dysfunction-based criteria. Future studies should evaluate whether the adhesion-molecule activation profile remains associated with pediatric sepsis when organ dysfunction-based definitions are used.

Third, microbiological data were not systematically collected. Blood cultures were performed only when clinically indicated by treating physicians and were not recorded as a standardized study variable. Therefore, we could not reliably classify infections as bacterial, viral, or culture-confirmed, nor could we report organism-level findings. This limitation is important because soluble adhesion-molecule elevation may reflect severe inflammatory activation more broadly, rather than sepsis-specific biology attributable to a particular pathogen group. To partly address clinical characterization, we reported available clinical and laboratory features, including CRP, lactate, white blood cell count, intensive care unit admission, and other cohort-level characteristics.

Fourth, biomarker concentrations were measured at a single time point, and temporal trajectories could not be assessed. Fifth, some analytes had zero values, which were handled as below-threshold values for log-transformed analyses. Although this below-threshold replacement strategy was prespecified, it may influence log-transformed analyses for markers with a high proportion of zero values. We therefore performed a sensitivity analysis excluding EpCAM and PSGL-1, which showed similar results. Sixth, the composite score, tertile-defined activation groups, and PCA-derived activation axis were derived and evaluated in the same cohort. These analyses are therefore exploratory and may be affected by cohort-specific correlation structure. Finally, the study did not include mechanistic cellular assays, so biological pathway interpretation remains inferential.

Future studies should validate these findings in larger multicenter pediatric cohorts, assess associations with organ dysfunction, shock, mortality, and length of stay, and compare simplified adhesion-marker approaches against routinely available markers such as CRP and clinical severity measures. Serial measurements may also help determine whether changes in soluble adhesion molecules over time improve risk stratification or reflect treatment response.

## 5. Conclusions

In this prospective Vietnamese pediatric cohort, children diagnosed with sepsis within 48 h of admission showed coordinated elevation of soluble adhesion molecules measured at enrollment. The composite 12-marker adhesion activation score and the principal component analysis-derived activation axis were strongly associated with sepsis, but they should be interpreted as exploratory summaries of correlated biomarker elevation rather than validated biological phenotypes. These findings support the biological relevance of leukocyte–endothelial activation in pediatric sepsis and provide a basis for future validation studies. Further work should determine whether simplified, clinically feasible adhesion-marker approaches can add value beyond C-reactive protein and routine clinical assessment.

## Figures and Tables

**Table 1:** Baseline characteristics by sepsis status.

Characteristic	Overall (*n* = 144)	Sepsis (*n* = 32)	Non-Sepsis (*n* = 112)	*p* Value
Age, months	17.3 (9.0–35.9)	11.0 (5.1–36.1)	18.8 (10.6–35.4)	0.100
Male sex, *n* (%)	89 (61.8)	19 (59.4)	70 (62.5)	0.837
Weight, kg	10.2 (8.1–13.0)	9.8 (7.0–13.2)	10.4 (8.6–13.0)	0.192
Gestational age, weeks	38 (38–39)	38 (38–39)	38 (38–39)	0.244
Temperature, °C	38.0 (37.4–38.7)	38.3 (37.4–39.2)	38.0 (37.4–38.5)	0.074
Heart rate, beats/min	157 (142–175)	165 (152–176)	155 (138–174)	0.125
Respiratory rate, breaths/min	40 (35–48)	42 (35–50)	40 (35–46)	0.215
SpO_2_, %	97 (96–99)	97 (96–99)	97 (96–98)	0.815
Hemoglobin, g/dL	11.2 (10.4–12.2)	10.9 (9.7–11.7)	11.3 (10.5–12.2)	0.092
White blood cell count, ×10^9^/L	11.8 (8.2–17.6)	14.4 (8.9–19.1)	10.6 (8.1–16.5)	0.131
CRP, mg/L	19.0 (3.9–63.1)	63.0 (14.4–113.1)	11.2 (2.8–31.1)	<0.001
Lactate, mg/dL	18.1 (14.4–24.4)	23.0 (17.0–31.4)	17.4 (13.9–22.4)	0.002
Glucose, mg/dL	103 (90–114)	103 (89–122)	103 (90–112)	0.727
ICU admission, *n* (%)	7 (4.9)	5 (15.6)	2 (1.8)	0.006
In-hospital death, *n* (%)	0 (0.0)	0 (0.0)	0 (0.0)	—

Data are presented as median (interquartile range) unless otherwise indicated. CRP, C-reactive protein; ICU, intensive care unit; SpO_2_, peripheral oxygen saturation.

**Table 2:** Concentrations of 12 soluble adhesion molecules by sepsis status.

Marker	Sepsis (*n* = 32), Median (IQR), ng/mL	Non-Sepsis (*n* = 112), Median (IQR), ng/mL	*p* Value
E-selectin	190.9 (36.9–222.4)	11.2 (0.9–34.3)	<0.001
P-selectin	3.4 (1.4–157.8)	1.0 (0.2–10.1)	0.001
sICAM-1	450.1 (148.6–862.0)	138.3 (13.4–272.6)	<0.001
sVCAM-1	2130.0 (1382.2–3721.5)	625.1 (147.9–880.5)	<0.001
CD44	14.2 (11.5–18.8)	10.8 (7.6–15.6)	0.003
PECAM-1	4.9 (3.0–6.8)	2.6 (1.7–3.6)	<0.001
EpCAM	0.1 (0.1–0.1)	0.0 (0.0–0.1)	<0.001
L-selectin	126.2 (85.2–277.4)	24.9 (8.8–56.1)	<0.001
ICAM-2	129.2 (43.8–220.2)	18.0 (4.1–35.5)	<0.001
PSGL-1	0.1 (0.0–0.1)	0.0 (0.0–0.0)	<0.001
NCAM	84.6 (50.1–161.5)	36.9 (11.2–74.7)	<0.001
ICAM-3	30.3 (16.1–46.7)	8.7 (4.9–13.1)	<0.001

Values are presented as median (interquartile range). CD44, cluster of differentiation 44; EpCAM, epithelial cell adhesion molecule; ICAM, intercellular adhesion molecule; IQR, interquartile range; NCAM, neural cell adhesion molecule; PECAM-1, platelet/endothelial cell adhesion molecule-1; PSGL-1, P-selectin glycoprotein ligand-1; sICAM-1, soluble intercellular adhesion molecule-1; sVCAM-1, soluble vascular cell adhesion molecule-1. For log-transformed analyses, zero values were treated as below-threshold values and replaced by one-half of the minimum positive value for the corresponding marker.

**Table 3:** Discriminatory performance of individual markers, composite score, PCA-derived axis, and CRP for sepsis within 48 h.

Marker or Score	AUC
L-selectin	0.883
sVCAM-1	0.855
PCA-derived adhesion activation axis, PC1	0.857
Composite adhesion activation score	0.855
ICAM-3	0.838
PSGL-1	0.836
E-selectin	0.819
ICAM-2	0.819
sICAM-1	0.773
PECAM-1	0.779
NCAM	0.749
CD44	0.672
P-selectin	0.689
CRP	0.698

AUC, area under the receiver operating characteristic curve; CRP, C-reactive protein; ICAM, intercellular adhesion molecule; NCAM, neural cell adhesion molecule; PC1, first principal component; PCA, principal component analysis; PECAM-1, platelet/endothelial cell adhesion molecule-1; PSGL-1, P-selectin glycoprotein ligand-1; sICAM-1, soluble intercellular adhesion molecule-1; sVCAM-1, soluble vascular cell adhesion molecule-1.

**Table 4:** Association of composite and PCA-derived adhesion activation measures with sepsis.

Variable	Odds Ratio	95% CI	*p* Value	AUC (95% CI)
Composite adhesion activation score, per 1-SD increase	7.95	3.44–18.40	<0.001	0.855 (0.776–0.931)
PC1 adhesion activation axis, per 1-SD increase	8.01	3.45–18.61	<0.001	0.857 (0.777–0.927)
Intermediate vs. low activation group	2.09	0.36–12.00	0.408	—
High vs. low activation group	27.18	5.91–124.95	<0.001	—

Odds ratios are reported per 1-standard deviation increase unless otherwise indicated. AUC confidence intervals were estimated using bootstrap resampling. AUC, area under the receiver operating characteristic curve; CI, confidence interval; PC1, first principal component; SD, standard deviation.

**Table 5:** Clinical characteristics across tertile-defined adhesion activation groups.

Characteristic	Low (*n* = 48)	Intermediate (*n* = 48)	High (*n* = 48)	*p* Value
Age, months	13.4 (9.2–35.0)	24.9 (13.1–40.5)	14.1 (5.7–35.9)	0.060
Male sex, *n* (%)	28 (58.3)	32 (66.7)	29 (60.4)	0.682
Weight, kg	10.1 (8.6–12.5)	11.0 (8.8–15.1)	10.2 (6.7–13.0)	0.159
Gestational age, weeks	38 (38–38)	38 (38–39)	38 (38–39)	0.118
Temperature, °C	38.0 (37.0–38.5)	38.0 (37.3–38.5)	38.3 (37.5–39.0)	0.132
Heart rate, beats/min	162 (141–175)	154 (138–177)	158 (146–174)	0.578
Respiratory rate, breaths/min	40 (35–50)	40 (35–43)	44 (35–48)	0.189
SpO_2_, %	97 (96–98)	97 (96–99)	97 (96–99)	0.306
Hemoglobin, g/dL	11.3 (10.6–12.0)	11.6 (10.8–12.2)	10.9 (9.8–12.2)	0.168
White blood cell count, ×10^9^/L	9.8 (7.8–15.9)	13.6 (9.6–21.3)	12.9 (8.5–18.3)	0.031
CRP, mg/L	9.8 (2.9–23.8)	16.6 (6.3–46.4)	28.7 (3.9–110.8)	0.066
Lactate, mg/dL	18.0 (13.6–23.2)	18.0 (14.3–23.5)	18.5 (15.5–25.2)	0.540
Glucose, mg/dL	105 (96–114)	102 (91–111)	99 (87–115)	0.240
Sepsis, *n* (%)	2 (4.2)	4 (8.3)	26 (54.2)	<0.001
ICU admission, *n* (%)	1 (2.1)	2 (4.2)	4 (8.3)	0.350

Data are presented as median (interquartile range) unless otherwise indicated. Activation groups were defined according to tertiles of the composite adhesion activation score, resulting in three equal-sized groups of 48 children. CRP, C-reactive protein; ICU, intensive care unit; SpO_2_, peripheral oxygen saturation.

## Data Availability

De-identified data may be made available from the corresponding author upon reasonable request and subject to institutional and ethical approval.

## Supplementary Materials

The following supporting information can be downloaded at: https://www.mdpi.com/article/10.3390/pediatric18030078/s1, Table S1: Detailed individual adhesion-marker ROC results for sepsis within 48 h; Table S2: Principal component loadings for the 12-marker adhesion panel; Table S3: Sensitivity analysis excluding EpCAM and PSGL-1; Figure S1: Spearman correlation matrix of 12 soluble adhesion molecules; Figure S2: PC1 loadings for the PCA-derived adhesion-molecule activation axis; Figure S3: Receiver operating characteristic curves for adhesion activation measures and leading individual markers.

## Related Manuscripts and Publication Transparency

This manuscript uses data from the same prospective cohort as related analyses addressing distinct clinical and methodological questions. The present manuscript focuses specifically on the 12-marker soluble adhesion-molecule panel, the composite activation score, and the PCA-derived adhesion-molecule activation axis. Related analyses will be disclosed to journal editors as required.

## Abbreviations

ALCAM	activated leukocyte cell adhesion molecule
AUC	area under the receiver operating characteristic curve
CD44	cluster of differentiation 44
CI	confidence interval
CRP	C-reactive protein
EDTA	ethylenediaminetetraacetic acid
EpCAM	epithelial cell adhesion molecule
ICAM	intercellular adhesion molecule
ICU	intensive care unit
IPSCC	International Pediatric Sepsis Consensus Conference
IQR	interquartile range
NCAM	neural cell adhesion molecule
PC1	first principal component
PCA	principal component analysis
PECAM-1	platelet/endothelial cell adhesion molecule-1
PSGL-1	P-selectin glycoprotein ligand-1
ROC	receiver operating characteristic
SD	standard deviation
sICAM-1	soluble intercellular adhesion molecule-1
sVCAM-1	soluble vascular cell adhesion molecule-1
STARD	Standards for Reporting Diagnostic Accuracy Studies
STROBE	Strengthening the Reporting of Observational Studies in Epidemiology
VCAM	vascular cell adhesion molecule
