# Supplementary material for: A Coordinated Adhesion-Molecule Activation Profile in Pediatric Sepsis: A Prospective Cohort Study from Vietnam
Source: Pediatr Rep. 2026 Jun 9;18(3):78. doi: 10.3390/pediatric18030078 (PMC13304983; doi:10.3390/pediatric18030078)
Supplement: Supplementary file 1 [file pediatrrep-18-00078-s001.zip › pediatrrep-4357321-supplementary.pdf]

Supplementary Appendix

A Coordinated Adhesion-Molecule Activation Profile in Pediatric Sepsis: A Prospective Cohort Study from Vietnam

This appendix contains supporting tables and figures that contextualize the composite adhesion activation score and the principal component analysis (PCA)-derived adhesion-molecule activation axis reported in the main manuscript.

Supplementary Table S1. Detailed individual adhesion-marker ROC results for sepsis within 48 hours

Marker	AUC (95% CI)	Youden cutoff, ng/mL	Sensitivity	Specificity
L-selectin	0.883 (0.827-0.932)	45.39	1.000	0.732
sVCAM-1	0.855 (0.761-0.940)	1254.86	0.812	0.938
ICAM-3	0.838 (0.732-0.915)	16.94	0.750	0.884
PSGL-1	0.836 (0.765-0.901)	0.04	0.750	0.804
E-selectin	0.819 (0.743-0.894)	112.67	0.688	0.884
ICAM-2	0.819 (0.717-0.901)	46.35	0.750	0.848
EpCAM	0.814 (0.730-0.894)	0.08	0.625	0.884
PECAM-1	0.779 (0.677-0.877)	5.32	0.500	0.955
sICAM-1	0.773 (0.670-0.858)	286.10	0.719	0.759
NCAM	0.749 (0.652-0.830)	42.41	0.844	0.536
P-selectin	0.689 (0.603-0.777)	1.06	0.875	0.509
CD44	0.672 (0.572-0.769)	9.95	0.906	0.473

Note. AUC confidence intervals were estimated using bootstrap resampling. AUC, area under the receiver operating characteristic curve; CI, confidence interval; ROC, receiver operating characteristic; sICAM-1, soluble intercellular adhesion molecule-1; sVCAM-1, soluble vascular cell adhesion molecule-1.

Supplementary Table S2. Principal component loadings for the 12-marker adhesion panel

Marker	PC1 loading	PC2 loading	PC3 loading
E-selectin	0.31	0.001	0.078
P-selectin	0.265	-0.368	-0.095
sICAM-1	0.316	-0.203	0.186
sVCAM-1	0.306	0.163	-0.23
CD44	0.257	-0.378	-0.445
PECAM-1	0.265	-0.161	0.569
EpCAM	0.31	-0.006	0.081
L-selectin	0.245	0.649	-0.238
ICAM-2	0.317	-0.073	-0.192
PSGL-1	0.258	0.334	0.483
NCAM	0.301	-0.156	0.025
ICAM-3	0.301	0.258	-0.204

Note. PCA was performed on standardized log2-transformed biomarker concentrations. PC, principal component.

Supplementary Figure S1. Spearman correlation matrix of 12 soluble adhesion molecules.

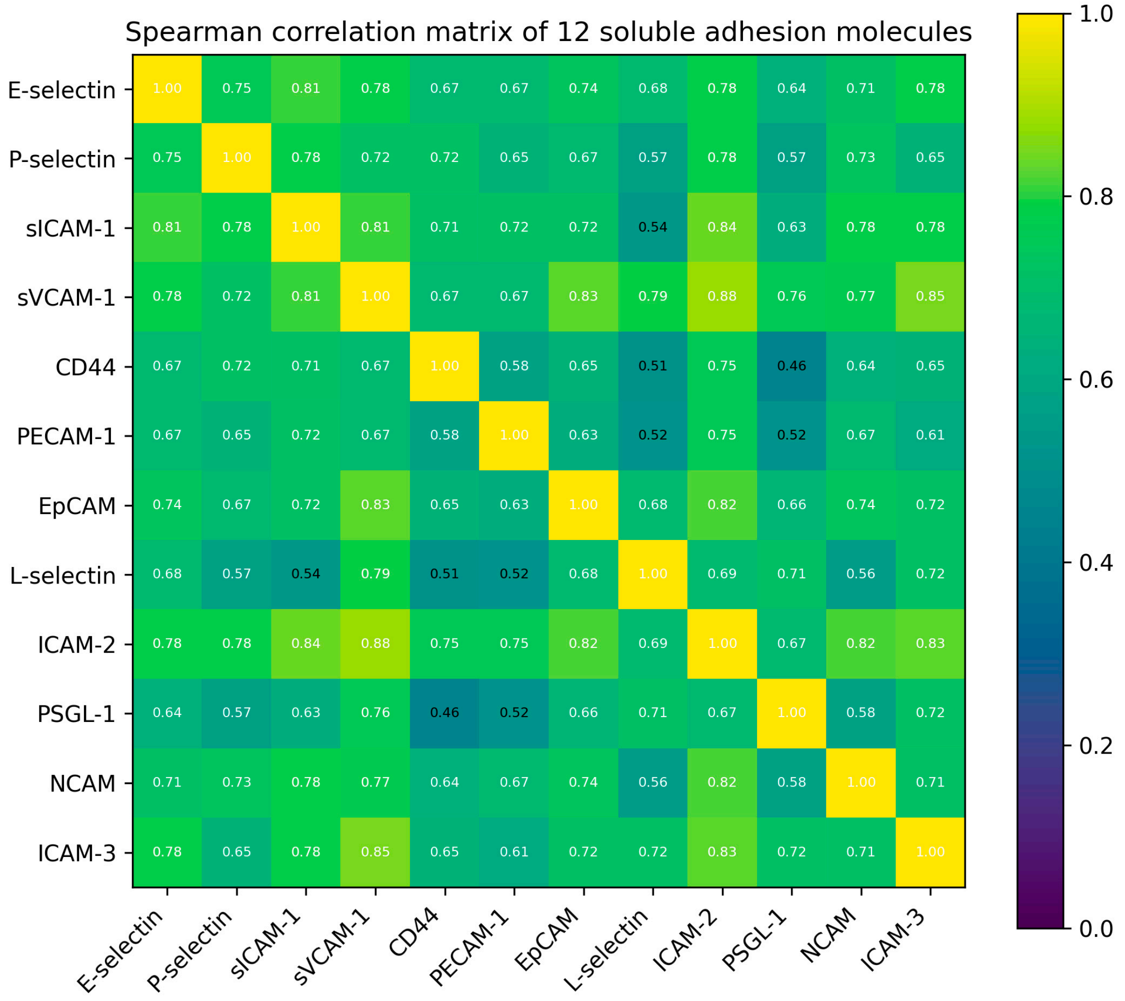

Supplementary Figure S2. PC1 loadings for the PCA-derived adhesion-molecule activation axis.

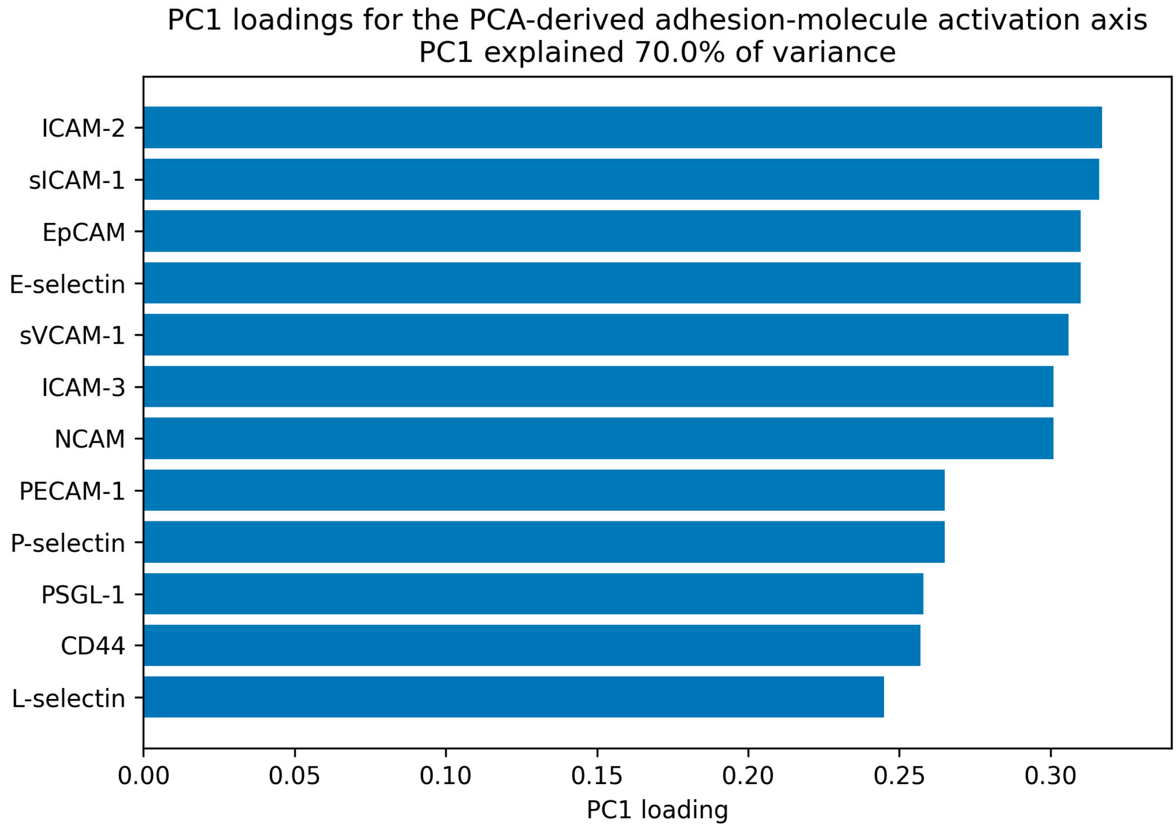

Supplementary Figure S3. Receiver operating characteristic curves for adhesion activation measures and leading individual markers.

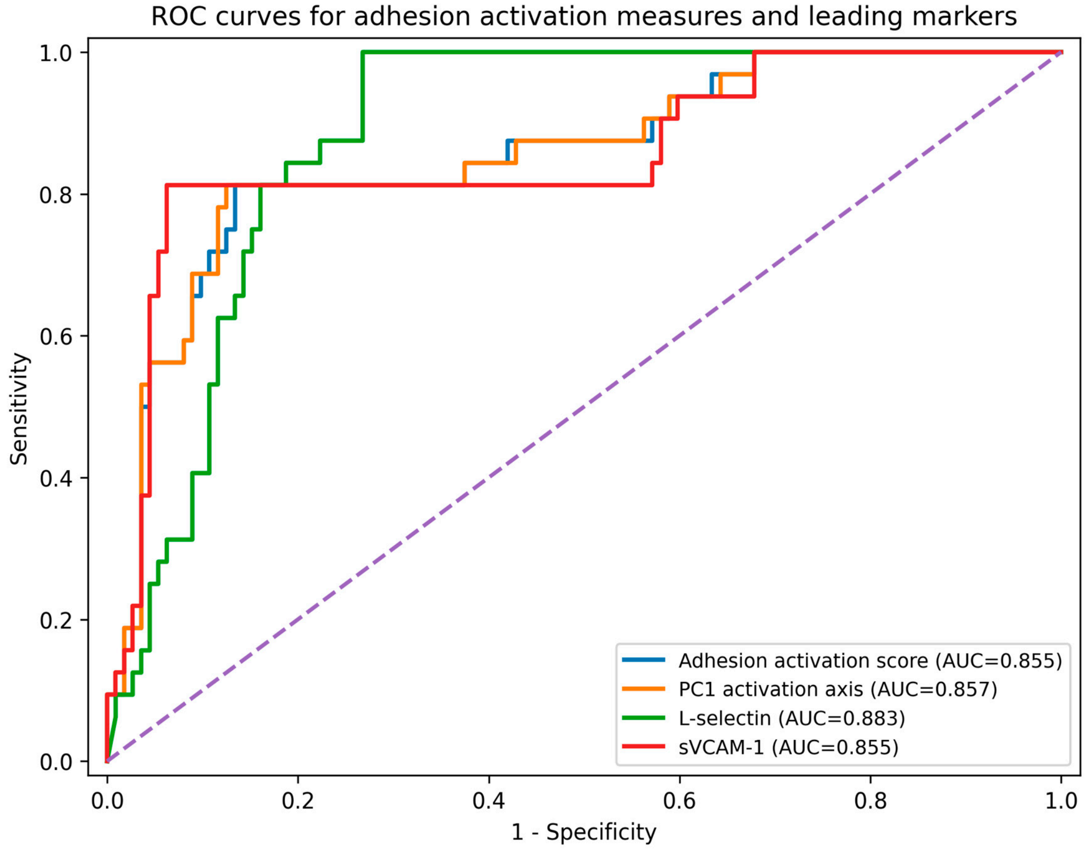

Zero-value handling: for log-transformed analyses, zero values were treated as below-threshold values and replaced by one-half of the minimum positive value for the corresponding marker before log2 transformation.

Supplementary Table S3. Sensitivity analysis excluding EpCAM and PSGL-1

Analysis	Odds ratio	95% CI	p value	AUC (95% CI)	PC1 variance explained
10-marker adhesion activation score, per 1-SD increase	6.35	3.01–13.38	<0.001	0.849 (0.762–0.924)	—
10-marker PC1 adhesion activation axis, per 1-SD increase	6.40	3.03–13.53	<0.001	0.848 (0.763–0.921)	70.9%

Note. The 10-marker sensitivity panel excluded EpCAM and PSGL-1 because these analytes contained zero values requiring below-threshold replacement before log2 transformation. The remaining markers were E-selectin, P-selectin, sICAM-1, sVCAM-1, CD44, PECAM-1, L-selectin, ICAM-2, NCAM, and ICAM-3. Odds ratios are reported per 1-standard-deviation increase. AUC confidence intervals were estimated using bootstrap resampling. PC1, first principal component; AUC, area under the receiver operating characteristic curve.
